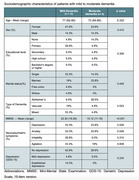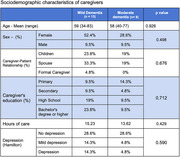# Perception of Quality of Life in patients with mild to moderate Dementia: A comparison between patients and caregivers evaluation

**DOI:** 10.1002/alz70858_103959

**Published:** 2025-12-26

**Authors:** Luis Raul Montiel‐Velazquez, Sandra Lizeth Celaya‐Hernández, Isaac Acosta‐Castillo, Mariana Longoria Ibarrola, Ana Luisa Sosa

**Affiliations:** ^1^ National Institute of Neurology and Neurosurgery, Tlalpan, DF, Mexico; ^2^ National Institute of Neurology and Neurosurgery, Mexico, Mexico; ^3^ National Institute of Neurology and Neurosurgery, Mexico City, Mexico; ^4^ Instituto Nacional de Neurología y Neurocirugía, Mexico City, DF, Mexico

## Abstract

**Background:**

“Quality of life” is defined as an individual's perception of their position in life according to cultural contexts and values. This concept represents one of the main priorities in the management of people living with dementia, modifying the perceived status of the disease, duration of care, neuropsychiatric symptoms, etc. Different questionnaires, applicable to patients and their caregivers, have been developed to assess quality of life. However, few studies compare the scores obtained between both of these subjects.

**Method:**

A cross‐sectional, observational, and comparative study was conducted on patients from the Cognitive Aging and Dementia Clinic diagnosed with Alzheimer's, Vascular, or Mixed dementia in mild or moderate stages and their caregivers. The study describes demographic variables and compares the scores obtained in the quality of life scales.

**Result:**

We obtained 21 responses from patients (61.9% and 38.1% with mild and moderate stage dementia) and their caregivers. The group with mild dementia had a mean age of 77 years (62‐90, *p* = 0.332), mostly female (47.6%, *p* = 0.410), and had mixed pathology (33.3%, *p* = 0.023). In the moderate dementia group, the mean age was 73 years (64‐85, *p* = 0.332), also primarily female (23.8%, *p* = 0.410), and the most frequent diagnosis was Alzheimer's (28.5%, *p* = 28.5%). The caregivers were mainly women, children, or spouses (42.8% and 52.5%, *p* = 0.676) and didn't have depression (*p* = 0.590). There were no significant differences in the total QoL‐AD score (34.1 in patients vs. 33.8 in caregivers, *p* = 0.880), except for self‐perception (*p* = 0.029) and economic status (*p* = 0.015), which were both higher for patients. Neither were there differences in the WHOQOL‐Bref questionnaire (81.19 in patients vs. 78.2 in caregivers, *p* = 0.502).

**Conclusion:**

Unlike previous studies, no significant differences were observed between the responses obtained from patients and caregivers when assessing quality of life. However, the scores for items related to self‐perception and economic status differed. Extending the study to other dementia syndromes or other care centers could strengthen and broaden the present study's analysis.